# Terahertz Sensing of L-Valine and L-Phenylalanine Solutions

**DOI:** 10.3390/s24123798

**Published:** 2024-06-12

**Authors:** Jingyi Shu, Xinli Zhou, Jixuan Hao, Haochen Zhao, Mingming An, Yichen Zhang, Guozhong Zhao

**Affiliations:** Beijing Key Laboratory for THz Spectroscopy and Imaging, Key Laboratory of THz Optoelectronics, Ministry of Education, Department of Physics, Capital Normal University, Beijing 100048, China; 2210602023@cnu.edu.cn (J.S.);

**Keywords:** terahertz, metasurface, sensor, glycerol, amino acid

## Abstract

To detect and differentiate two essential amino acids (L-Valine and L-Phenylalanine) in the human body, a novel asymmetrically folded dual-aperture metal ring terahertz metasurface sensor was designed. A solvent mixture of water and glycerol with a volume ratio of 2:8 was proposed to reduce the absorption of terahertz waves by reducing the water content. A sample chamber with a controlled liquid thickness of 15 μm was fabricated. And a terahertz time-domain spectroscopy (THz-TDS) system, which is capable of horizontally positioning the samples, was assembled. The results of the sensing test revealed that as the concentration of valine solution varied from 0 to 20 mmol/L, the sensing resonance peak shifted from 1.39 THz to 1.58 THz with a concentration sensitivity of 9.98 GHz/mmol∗L^−1^. The resonance peak shift phenomenon in phenylalanine solution was less apparent. It is assumed that the coupling enhancement between the absorption peak position of solutes in the solution and the sensing peak position amplified the terahertz localized electric field resonance, which resulted in the increase in frequency shift. Therefore, it could be shown that the sensor has capabilities in performing the marker sensing detection of L-Valine.

## 1. Introduction

Amino acids can be categorized as essential and non-essential based on their ability to be synthesized in the human liver. Measuring the levels of essential amino acids in the body can assist in diagnosing certain diseases, such as liver dysfunction, kidney dysfunction, hyperthyroidism, chronic malnutrition, and so forth. Essential amino acids play a crucial role in the proper functioning of various bodily processes [1,2,3]. Both L-Valine and L-Phenylalanine are essential amino acids in the human body and serve as key raw materials for protein synthesis. L-Valine maintains the function and structural stability of liver cells and can also provide energy for physical activity through oxidative metabolism. L-Phenylalanine is a precursor for melanin and various neurotransmitters, affecting skin color and reflecting neural activity. The detection and differentiation of these two amino acids in the human body are crucial [4,5].

The current mainstream biochemical sample testing on the market requires labeling of the samples being tested [6,7,8,9]. This labeling method is complex and time-consuming and can damage the tested samples. These factors restrict the application scope and testing accuracy of labeled sensors, which makes it crucial to seek a rapid and non-destructive sensing detection method.

The terahertz frequency range has great potential in fields such as chemistry and the detection of biological substances because it is characterized by low photon energy, broad band characteristics, and specific “fingerprint spectra” for the detection of biochemical macromolecules [10,11,12,13,14,15,16]. However, the resolution of the traditional THz-TDS technique is limited, and its sensitivity is not sufficient to detect differences in the spectral characteristics of trace samples. Therefore, in order to achieve the sensor detection of trace samples, it is necessary to enhance the intensity of the THz wave locally so that the requirements for the samples can be lowered.

Electromagnetic metasurfaces are composed of periodically arranged subwavelength unit cells. Extraordinary properties, unattainable by natural materials, are exhibited by them [17,18,19,20]. Designing special geometries on the metasurface can correspond to the strong absorption of terahertz waves at certain frequencies, which is also correlated with the near-surface refractive index and dielectric constants. When different concentrations or types of solutions are coated onto the designed metasurface, there will be slight variations in its surface refractive index and dielectric constants, leading to a frequency shift in the absorption peak position. Due to the frequency problem of the microwave band, it is difficult to improve the quality factor and sensitivity of the sensor. Increasing the sensor’s applicable frequency to the terahertz band can significantly increase sensitivity [21,22]. Zhang et al. proposed a metamaterial biosensor that achieves polarization-independent electromagnetic induction transparency (EIT) at terahertz frequencies. By growing two types of glioma cells (mutant and wild type) on the surface of a biosensor, mutant and wild-type glioma cells can be directly distinguished by observing changes in the EIT resonance frequency and amplitude at arbitrary cell concentrations without the introduction of antibodies [23]. Metasurfaces designed based on this feature serve in terahertz wave bands as sensors with higher detection sensitivity, without the need for labeling, and with time-saving and low cost, offering unique advantages, with them being particularly attractive for biomedical and chemical sensing platforms [24,25,26].

However, so far, the progress of experiments on metasurface sensing in the terahertz waveband has remained at the stage of sensing tests with different proportions of mixed solutions and testing on the dried samples after spraying or incubating samples. Chen et. al. proposed a Fano asymmetric split ring resonator sensor to culture protein A/G and protein A/G + protein lgG, and the results showed that the resonant peaks of the sensor covered with two proteins have different frequency shifts (8.9 GHz and 17.6 GHz) [27]. A metamaterial-based terahertz biosensor experiment was used to measure the correlation experiments of cancer cells, and the experimental results showed that the resonance frequency would redshift as the concentration of cancer cells increased. And they also used biosensors to study the effects of different drug concentrations on the apoptosis of cancer cells HSC3. The results show that this trend is consistent with the results of the biological CCK-8 kit method, which provides a valuable supplementary reference for biological research [28]. Chen et al. designed a metasurface sensor based on the Fano resonance principle to detect different proportions of water and methanol mixed solutions, which provides a reliable way for high-precision concentration detection in the field of chemistry [29]. Forouzeshfard et al. designed an optical semiconductor metamaterial sensor for NaCl and ethanol solute concentration sensing, and they obtained the value of 77 µm∗dm^2^/mol for the sensitivity of a NaCl solution sensor and the value of 87.7 GHz/RIU for ethanol–water solution [30]. Yang et al. designed a gold split ring resonator and used THz-TDS to detect the genomic DNA of transgenic tomato [31]. This is primarily due to the strong polarity of water solutions, which severely inhibits terahertz waves, and the absorption characteristics of solutes are also severely inhibited, which puts forward the requirement that the tested materials are supposed to be thin enough or even dehydrated. Moreover, recent works often focus on pursuing the sensing performance of the sensor itself, as well as requiring consideration of the differentiation and identification of different substances in sensing experiments [32,33,34].

In this paper, an asymmetrically folded dual-aperture metal ring terahertz metasurface sensor was designed and optimized. A solvent mixture of water and glycerol in a volume ratio of 2:8 was proposed to reduce the water absorption of terahertz waves, thus avoiding the disadvantage of uneven solute distribution caused by methods such as drying evaporation or spraying. In order to achieve the optimal thickness of the test solution and maintain the same thickness from test to test, a sample chamber with a controlled liquid thickness of 15 μm was fabricated. We constructed a vertical incidence THz-TDS for the easy horizontal placement of the chamber. Subsequent sensing tests were conducted on solutions containing trace amounts of L-Valine and L-Phenylalanine. The experimental results revealed a pronounced frequency shift in the metasurface resonance peak with the increase in L-Valine solution concentration, showing a concentration sensitivity of $9.98\;GHz/mmol*L^{-1}$. Similarly, an increase in the concentration of the L-Phenylalanine solution resulted in a lower frequency shift, with a concentration sensitivity of $1.88\;GHz/mmol*L^{-1}$.

## 2. Simulation Design and Experimental Methods

**Figure 1:**
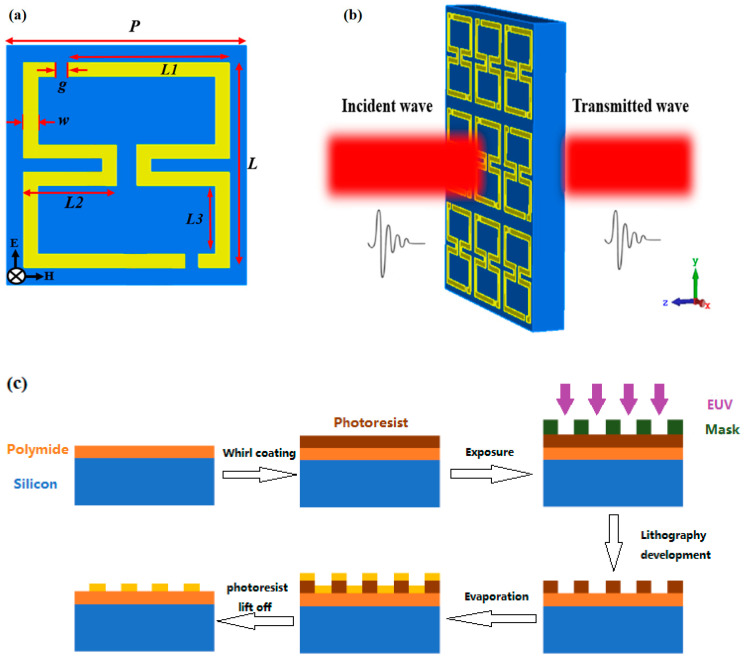
Metasurface sensor’s (**a**) unit structure schematic; (**b**) working principle diagram; and (**c**) Lithography process.

An asymmetrically folded dual-aperture metal ring terahertz metasurface sensor was designed. The schematic diagram and principle of the sensor unit structure are illustrated in Figure 1a,b [35]. The bottom layer is polyimide (PI), with a thickness of 35 μm, and the top layer is gold, with a thickness of 0.2 μm. The structural dimensions include a unit cell period P=97 μm, L=80 μm, L1=63 μm, L2=37 μm, L3=31 μm, line width w=6 μm and opening gap g=6 μm; the terahertz wave propagates along the negative *z*-axis, and the electric field direction is along the *y*-axis. The electromagnetic simulation software CST Studio Suite 2020 was used to perform the simulation calculations using the finite–difference time-domain (FDTD) method. The preparation of the metasurface was carried out by using photolithography at the 13th Research Institute of China Electronics Technology Corp (Shijiazhuang, China). AZ5214 reverse adhesive was selected for the photoresist, and its positive adhesive properties were utilized. The mask pattern is the opposite of the required structural pattern. When UV light shines on the photoresist through the non-shading part of the mask, the photosensitive agent in the photoresist will decompose and become a solubility enhancer. The photoresist here is readily soluble in the alkaline developer, and no chemical changes occur in the shading area. A gold film was plated on the substrate surface via vacuum evaporation. In order to increase the adhesion between the gold film and the photoresist, a layer of chromium film must be plated before gold plating. After gold plating, the residual photoresist is cleaned off to form the desired metal structure. The preparation process is shown in Figure 1c.

Q-factor (Q) and sensitivity (S) are critical parameters to evaluate the performance of metasurface sensors. As shown in Formula (1), the Q-factor can be used to characterize the resolution of metasurface sensors, and it can be defined as the ratio of the center frequency $f_{0}$ of the resonant peak to the full width at half maximum (FWHM). The larger Q, the sharper the resonance peak.

(1) $$Q=\frac{f_{0}}{FWHM}$$

The sensitivity (S) of a sensor is the degree of change in the sensor caused by a unit change in the sample. When the unit change is the refraction index shift (∆n) and it causes the resonant frequency shift (∆f), sensitivity can be calculated using Formula (2):

(2) $$S=\frac{∆f}{∆n}$$

When the unit change is the solution concentration shift (∆c) and it causes the resonant frequency shift (∆f), sensitivity can be calculated using Formula (3):

(3) $$S=\frac{∆f}{∆c}$$

**Figure 2:**
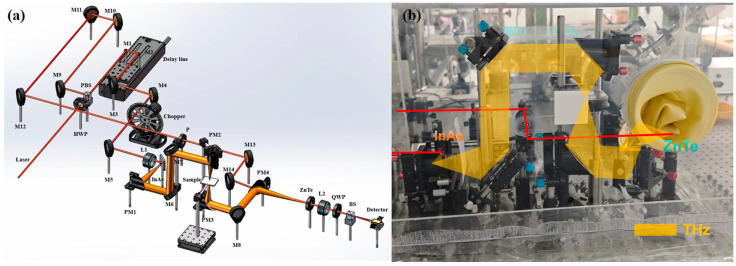
Vertical incident THz-TDS: (**a**) optical path and (**b**) system diagram.

A vertical incidence THz-TDS system was set up. The laser used in the laboratory is the titanium gem femtosecond laser (Chameleon Ultra II, Coherent Inc., Santa Clara, CA, USA); the total optical power is 1.6 W, the repetition rate is 80 MHz, and the pulse duration is 100 fs. A femtosecond laser is used to irradiate the surface of semiconductor indium arsenide (InAs) to generate terahertz radiation, and electro-optic sampling (EOS) is used for detection, as depicted in Figure 2a,b, to place the liquid samples horizontally. Compared with the traditional THz-TDS system, this system is more suitable for the sensing and detection of solution samples, which ensures that the solution samples remain uniformly distributed during the measurement process, thus ensuring the accuracy of the experimental results. The system has a minimum spectral resolution of 9 GHz, a dynamic range of 2950, and a signal-to-noise ratio (SNR) of 645.

In order to further reduce the absorption of terahertz waves by water vapor in the air, the terahertz optical path was covered by a self-made glove box with acrylic boards, continuously filled with dry air to maintain a relative humidity of 8% inside the box. In order to facilitate the placement and replacement of samples, a square hole with a size of 40 mm∗40 mm on the glove box was cut, and the same-sized square soft plugs blocked the hole to ensure good sealing of the glove box during the progression of the experiment. This allowed for easier replacement of the samples.

**Figure 3:**
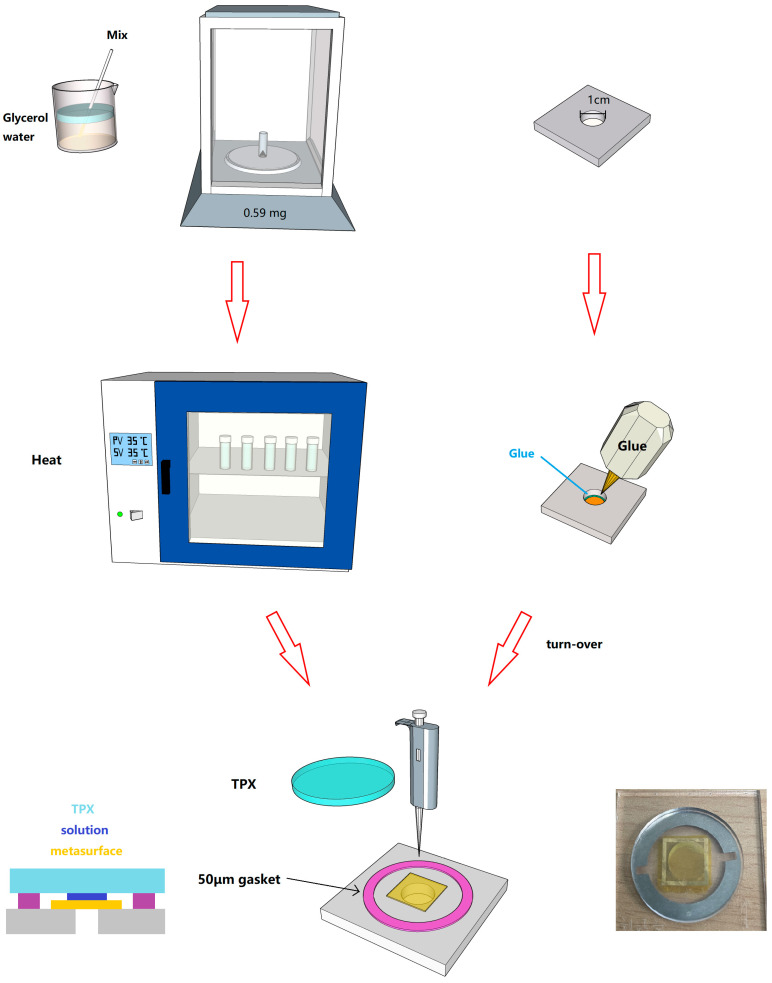
Experimental preprocessing diagram.

The preparation of samples and chamber schematics are shown in Figure 3. Before the experiment, in order to ensure that the sensor surface was flat and the thickness of the covering solution was consistent, a fixed-thickness microfluidic chip fabrication method was proposed. A 40 mm∗40 mm∗3 mm acrylic plate was taken and a circular hole with a straight well of 10 mm was opened in the center, and the sensor structure was placed with the structural side of the sensor facing downwards and the PI substrate facing upwards. The acrylic plate was placed on the sensor and flattened, and the open hole exposed the sensor’s working area. We applied a small amount of glue to the contact between the PI substrate and the acrylic plate. To apply the glue, we used a thickness gauge to measure the thickness of the bond; the test results showed the average thickness to be 35.035 mm, indicating that the glue rarely penetrated between the acrylic plate and the sensor, which did not result in an increase in thickness. A batch of stainless-steel ring spacers with a thickness of 50 μm was then customized to develop the cover layer thickness, with a 2 mm high top cover made of 4-methylpentene-1 polymer (named TPX). By using this microfluidic chip, the thickness of the tested solution was fixed at 15 μm.

Solutions with 0, 5, 10, and 20 mmol/L concentrations were prepared and subjected to sensing tests. After configuring the solution, it was uniformly placed in a drying oven at 35 °C and heated for two hours to accelerate solute dissolution. The detection position of this system was the focus spot, whose diameter was around 1.2 mm. To ensure optimal sensing efficiency, we used a solution column diameter of 10 mm and a height of 15 μm. According to the calculation, by using a pipette, the appropriate amount to be dropped into the center of the metasurface was 2.5 μL. As for sample pool cleaning, we used a cleaning method with alcohol–acetone–alcohol to ensure no solution residue.

## 3. Results and Discussion

### 3.1. Simulation Analysis and Characterization of the Metasurface

**Figure 4:**
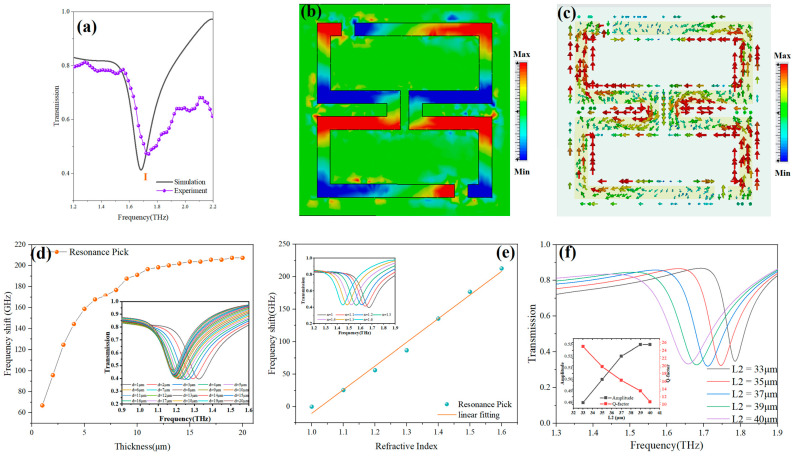
Asymmetric folded double open ring’s (**a**) experimental characterization and simulation comparison; (**b**) surface electric field distribution; (**c**) surface current distribution; (**d**) the influence of the thickness of the cover on the frequency shift of the resonant peak; (**e**) the influence of the change in refractive index on the resonant peak frequency shift; and (**f**) the effect of L2 size on sensor performance.

The experimental and simulated transmission spectra of the sensor are presented in Figure 4a. The resonant frequency at peak I in the simulation model was 1.690 THz with Q=16.28, while during experimental testing, it was observed at 1.719 THz and Q=9. The experimental results are in agreement with the simulation results.

To determine the resonance mechanism of metasurface sensors, the analysis of the surface current and electric field distribution at 1.690 THz is shown in Figure 4b,c. Four pairs of dipole resonances are observed on either side of the folded aperture ring, with a high-intensity surface electric field indicating strong absorption of terahertz waves and a characteristic of dipole resonance modes. Moreover, the effects of solution coverage thickness and different solution concentrations on sensor performance were also investigated. Setting the refractive index of the solution to n = 2, the solution thickness was varied from 1 μm to 20 μm at intervals of 1 μm. The relationship between solution thickness and peak shift at the resonant frequency corresponding to n = 1 is plotted in Figure 4d. With the increase in solution thickness, the sensing performance of the terahertz metasurface sensor gradually improved. At the solution thickness of 15 μm, the peak shift approached its maximum. Hence, the solution thickness was maintained at 15 μm in the experiments. After the solution thickness was controlled at 15 μm, the refractive index was varied, and its change was fitted against the peak shift of the sensor, yielding a refractive index sensitivity of 370 GHz/RIU, as shown in Figure 4e. The influence of the structure’s size on the performance of the sensor was investigated. The increase in L2 will lead to an increase in the quality factor of the resonant peak, but it will also reduce the amplitude of the resonant peak, as shown in Figure 4f. The size of L2 = 37 μm after comprehensive consideration. Through a series of design and optimization procedures, we obtained a terahertz metasurface sensor with excellent performance for the study of trace amino acid concentrations.

### 3.2. Selection of Different Volume Ratios of Water–Mixed Glycerol Solvent

**Figure 5:**
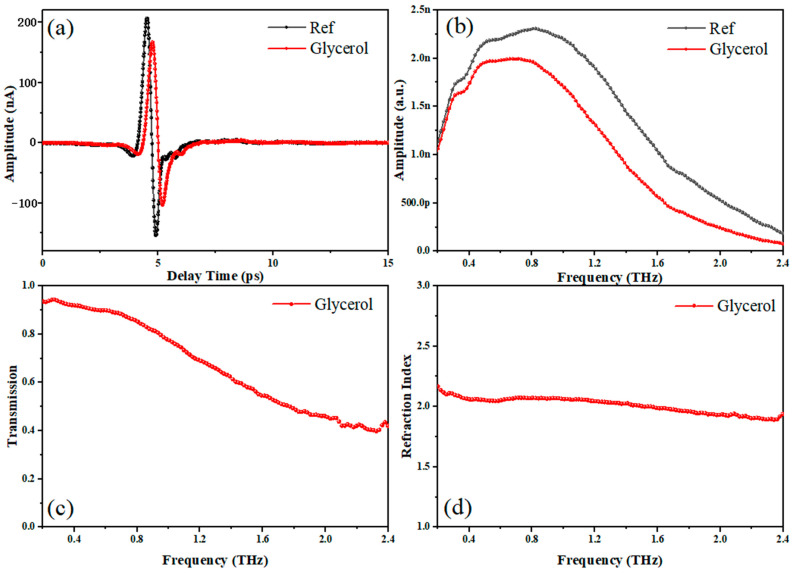
Terahertz spectrum of glycerol: (**a**) time-domain spectroscopy; (**b**) frequency domain spectrum; (**c**) transmission spectrum; (**d**) refractive index spectrum.

It is known that water exhibits strong absorption in the terahertz range. When using water as a solvent to detect trace substances in the terahertz band, it is necessary to consider whether the fingerprint spectrum of the substance will be submerged in the aqueous solution and whether too thick an aqueous solution will cause the spectrum width to decrease. The working principle of a terahertz metasurface sensor is that the refractive index and dielectric constant of the near surface change due to the change in substance concentration or mass, which causes the resonant peak frequency shift. However, current terahertz metasurface sensors often require a sufficiently 10 μm thick cover layer to ensure their sensing performance [36]. Using polar solvents to carry out mixing with water can reduce the absorption of terahertz waves by water while ensuring a certain solubility. A method of mixing glycerol with distilled water in proportion as an alternative to a pure water cover has been proposed. Glycerol, as a polar solvent, has advantages such as low biological toxicity, arbitrary compatibility with water in any ratio, and a certain ability to dissolve biological molecules [37,38]. The frequency domain, transmission, and refraction index spectrum of glycerol are shown in Figure 5. Despite being a polar solvent, glycerol exhibits good transparency in the terahertz range and shows no significant absorption peaks in the 0.2–2.4 THz frequency range.

**Figure 6:**
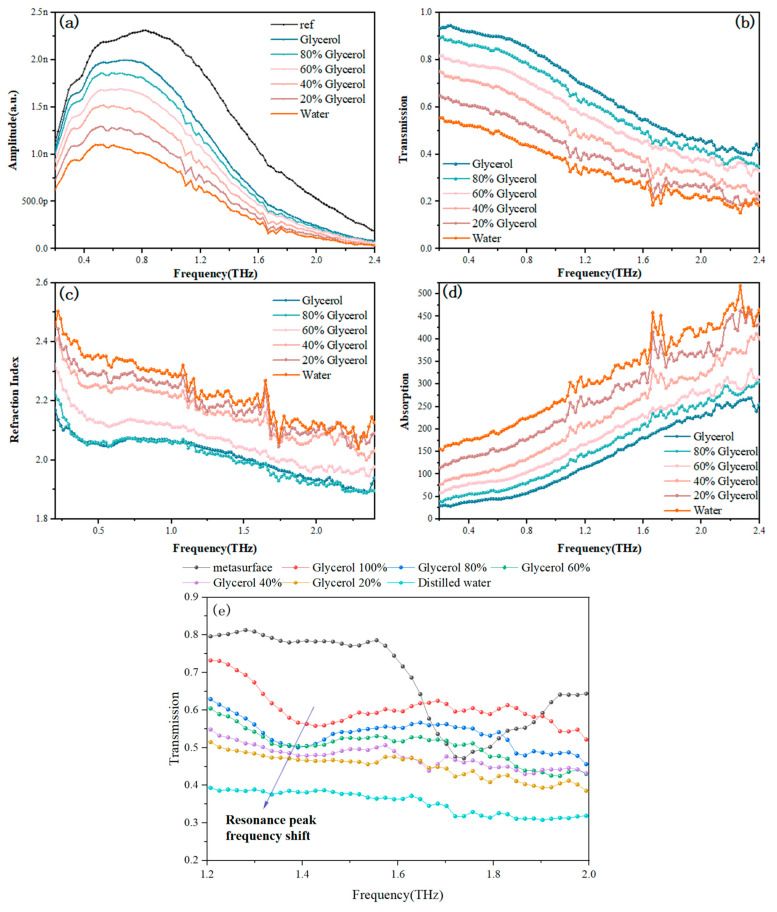
Terahertz spectrum of solutions of water mixed with glycerol in different proportions (**a**) frequency domain spectrum; (**b**) transmission spectrum; (**c**) refractive index spectrum; (**d**) absorption spectrum; (**e**) transmission spectrum of the sensor with mixed solutions.

Based on the good absorption properties of glycerol in the terahertz band, this paper proposes replacing the aqueous solvent with glycerol mixed with water, so as to reduce the influence of the solvent on the sensing test [39]. Here, the solvent mixtures of different volume ratios of water and glycerol were produced. The 50 μm thick mixture solvent was tested first, and then the 15 μm thick mixture solvent was covered on the metasurface for sensing detection. The results are shown in Figure 6. As the volume ratio of distilled water in the solvent increases, there is a gradual decrease in the transmittance of the solution with a transmittance depression, and the transmission depression of the water solution appears, with a rise in the refractive index accompanied by a certain refractive anomaly, and a gradual increase in the absorptivity with an absorption peak was observed. Similarly, the mixed solution was tested after covering the terahertz metasurface sensor, and it was observed that the resonant peaks modulated by the sensor gradually redshift and are suppressed. The overall transmittance decreases gradually. And when the covering layer is pure water, it is impossible to distinguish resonant peaks at high frequencies, which indicates that water absorption significantly inhibits the modulated resonant peaks of the metasurface, making it difficult to identify sensing peaks. Based on this experiment, in order to ensure higher transmittance and certain solubility at the same time, a water and glycerol volume ratio of 2:8 as the solvent was chosen.

### 3.3. Sensing Experiment

**Figure 7:**
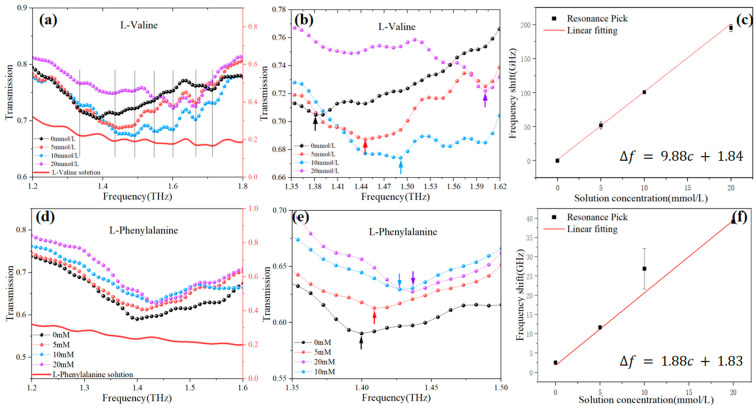
(**a**) Transmission spectrum of L-Valine solution; (**b**) the optimized transmission spectrum of L-Valine solution; (**c**) fitting analysis of L-Phenylalanine sensing frequency shift and solution concentration; (**d**) transmission spectrum of L-Phenylalanine solution; (**e**) the optimized transmission spectrum of L-Valine solution; (**f**) fitting analysis of L-Phenylalanine sensing frequency shift and solution concentration.

Before the experiment, 2.34 mg and 1.17 mg of L-Valine and 3.30 mg and 1.65 mg of L-Phenylalanine were dissolved in a 1 mL solution of water mixed with glycerol with a volume ratio of 2:8. In this way, the L-Valine and the L-Phenylalanine solutions with a concentration of 20 mmol/L and 10 mmol/L could be matched. As for a solution with a concentration of 5 mmol/L, it is too difficult and inaccurate to weigh 0.585 mg manually. The dilution method was used to mix 500 μL of 10 mmol/L concentration solution with 500 μL of solvent. Figure 7a presents the transmission spectra detected on the metasurface covered with solutions of different concentrations of L-Valine. It was observed that with an increase in solute concentration, the resonance troughs of the sensor gradually blue-shifted. The bold red line in the graph represents the transmission spectrum of the L-Valine solution, where the transmission notches correspond to the weak absorption of the solid pellets and the jitter observed at the same frequency during the sensor experiments. From this, it is assumed that the peaks of the jitter observed during the sensor experiments are caused by the solute in the L-Valine solution, and the significant amplitude of the jitter observed during sensor experiments is evidently the result of modulation amplification by the sensor. The spectrum shown in Figure 7b was obtained by eliminating the influence of the solute in the sensor detection spectrum results. The experimental spectral resolution in this paper is 9 GHz. The frequency shifts were fitted and analyzed against the solution concentrations, yielding a linear relationship of ∆f=9.98c+1.84, with a concentration sensitivity of $S=9.98\;GHz/mmol*L^{-1}$ (Figure 7c).

Similarly, sensing experiments were conducted on L-Phenylalanine. Figure 7d illustrates the sensing detection results of the L-Phenylalanine solution. With an increase in solute concentration, there is a smaller blue shift in the resonance troughs of the sensor. The bold red line on the graph represents the transmission spectrum of the L-Phenylalanine solution. A comparison revealed that the transmission jitter of the L-Phenylalanine solution at 1.2–1.6 THz was smaller, and the jitter observed during sensor experiments was also smaller, reflecting different characteristics compared to L-Valine solution sensing. The spectrum shown in Figure 7e was obtained by eliminating the influence of the solute in the sensor detection spectrum results. The frequency shifts were fitted and analyzed against the solution concentrations, yielding a relationship of ∆f=1.88c+1.83 (Figure 7f).

**Figure 8:**
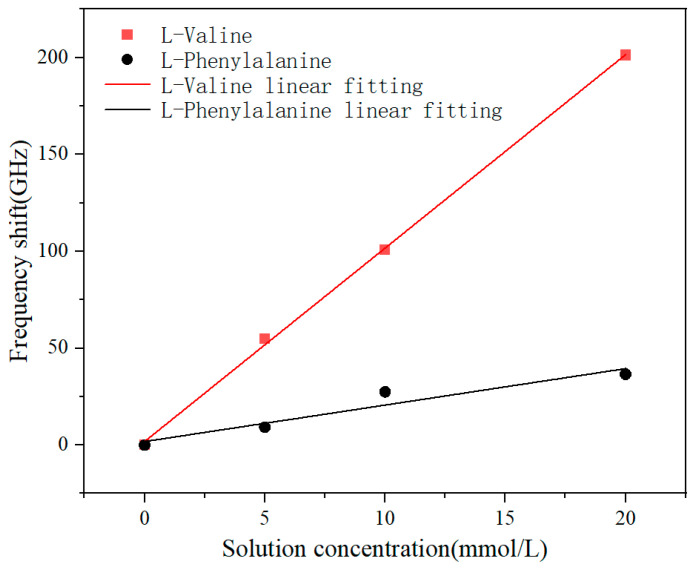
Two amino acid sensing frequency shifts and solution concentration fitting comparison.

The comparative analysis of the frequency shifts obtained from the sensing experiments on solutions of two amino acids is presented in Figure 8. The metasurface exhibits a pronounced response to variations in the concentration of L-Valine solution while displaying a weaker blue shift in response to L-Phenylalanine solution. It was assumed that the coupling between the absorption peaks of the solutes and the resonant peaks of the sensor design significantly enhanced the sensing capability, thereby achieving specificity in sensing during the experimental setup.

The experimental results show that the resonance peak has a blue shift phenomenon. Compared to simulation analysis and the experiment that involved drying the solution, it can be inferred that adding a small amount of amino acids to the mixed solution of water and glycerol in the volume ratio of 2:8 cannot increase the refractive index of the solution but instead decreases it. It is inferred that the presence of a large amount of glycerol in the mixed solution inhibits the hydrogen bond of water, and the addition of a small amount of amino acids intensifies this inhibition.

It can be seen from the experiments in this paper that using a mixed solution as a solvent can effectively reduce the absorption of terahertz waves by water, thus avoiding the uneven distribution of solute caused by drying solution on the metasurface. But this approach also has some limitations. Considering that the solubility of amino acids in water is in the range of 1620.2 (L-Proline) to 5 (Aspartic acid), when mixing solvents, a low proportion of water means low solubility, which results in the solute being insoluble at concentrations above 100 mmol/L.

## 4. Conclusions

In this study, an asymmetrically folded dual-aperture metal ring terahertz metasurface sensor was designed and optimized. Through simulation, the resonant peak was at 1.70 THz, the thickness of the covering solution was 15 microns, and the refractive index sensitivity was up to 370 GHz/RIU. A preparation method for a sample pool with controlled liquid thickness was proposed for solution sensing detection. Different volume ratios of water and glycerol mixed solutions were tested, and the ratio of 2:8 water and glycerol mixed solution was selected as the solvent to reduce the water content and thus reduce the absorption of THz waves by the solvent. Finally, different concentrations of L-Valine and L-Phenylalanine solutions were detected, and the detection results showed a prominent sensing phenomenon for L-Valine ($S=9.98\;GHz/mmol*L^{-1}$) while a weaker one for L-Phenylalanine ($S=1.88\;GHz/mmol*L^{-1}$). It was assumed that the enhancement of sensing was attributed to the coupling between solute absorption peaks and sensor resonance peaks; thus, the specificity of sensing was achieved.

## Data Availability

The datasets generated and analyzed during the current study are available from the corresponding author upon reasonable request.
